# Synaptic plasticity in two cell types of central amygdala for regulation of emotion and pain

**DOI:** 10.3389/fncel.2022.997360

**Published:** 2022-10-26

**Authors:** Jianlong Ge, Youqing Cai, Zhizhong Z. Pan

**Affiliations:** Department of Anesthesiology and Pain Medicine, The University of Texas MD Anderson Cancer Center, Houston, TX, United States

**Keywords:** amygdala circuits, synaptic plasticity, glutamate, pain, emotion

## Abstract

The amygdala is a critical brain site for regulation of emotion-associated behaviors such as pain and anxiety. Recent studies suggest that differential cell types and synaptic circuits within the amygdala complex mediate interacting and opposing effects on emotion and pain. However, the underlying cellular and circuit mechanisms are poorly understood at present. Here we used optogenetics combined with electrophysiological analysis of synaptic inputs to investigate pain-induced synaptic plasticity within the amygdala circuits in rats. We found that 50% of the cell population in the lateral division of the central nucleus of the amygdala (CeAl) received glutamate inputs from both basolateral amygdala (BLA) and from the parabrachial nucleus (PBN), and 39% of the remaining CeAl cells received glutamate inputs only from PBN. Inflammatory pain lasting 3 days, which induced anxiety, produced sensitization in synaptic activities of the BLA–CeAl–medial division of CeA (CeAm) pathway primarily through a postsynaptic mechanism. Moreover, in CeAl cells receiving only PBN inputs, pain significantly augmented the synaptic strength of the PBN inputs. In contrast, in CeAl cells receiving both BLA and PBN inputs, pain selectively increased the synaptic strength of BLA inputs, but not the PBN inputs. Electrophysiological analysis of synaptic currents showed that the increased synaptic strength in both cases involved a postsynaptic mechanism. These findings reveal two main populations of CeAl cells that have differential profiles of synaptic inputs and show distinct plasticity in their inputs in response to anxiety-associated pain, suggesting that the specific input plasticity in the two populations of CeAl cells may encode a different role in amygdala regulation of pain and emotion.

## Introduction

The amygdala is a key limbic structure for regulation of the brain’s emotional responses, including negative emotions such as anxiety, depression and pain, and positive emotions in reward (Janak and Tye, 2015; de Guglielmo et al., 2017; Wang et al., 2017; Nation et al., 2018; Neugebauer et al., 2020; Samineni et al., 2021). Pain is a common neurological disorder that involves both sensory component of nociception and affective component of negative emotion including anxiety and depression (Neugebauer, 2015; Duenas et al., 2016). While the brain’s cortical circuits appear more involved in integration, evaluation and motor responses of sensory and cognitive features of pain, the limbic amygdala plays a major role in pain-emotion association and memory consolidation through converged inputs from nociceptive spinothalamic pathways and affective/cognitive corticolimbic pathways (Vogt, 2005; Shackman et al., 2011; Baliki and Apkarian, 2015; Han et al., 2015; Neugebauer, 2015). However, how pain stimulus induces emotion changes and how pain sensation and emotion affect each other on the brain’s regulating circuits remain largely unknown. The critical involvement of the amygdala in regulation of both pain and emotion provides an optimal brain substrate for investigation of underlying circuit mechanisms on this topic.

Within the amygdala circuits, the current understanding of the major information flow is along the pathway of basolateral amygdala (BLA)—lateral division of the central nucleus of the amygdala (CeAl)—medial division of the central nucleus of the amygdala (CeAm). BLA receives major inputs from cortical structures and acts as a hub for communication between cortical structures and amygdala (McCool et al., 2010; Etkin et al., 2011). CeAl receives predominant glutamate inputs both from BLA and from the parabrachial nucleus (PBN) in the dorsolateral pons that carries peripheral pain signals, whereas CeAm receives prevailing GABAergic inputs from CeAl and functions as the major output of the amygdala complex (Baxter and Murray, 2002; Gottfried et al., 2003; Murray, 2007; Tye et al., 2011; Mahan and Ressler, 2012; Fernando et al., 2013; Cai et al., 2018; Neugebauer et al., 2020). Thus, CeAl appears the primary amygdala site for pain-emotion integration and interaction.

Previous optogenetic studies have demonstrated causal roles of the BLA–CeAl–CeAm circuit in regulation of anxiety behavior. For example, optical activation of the BLA–CeAl pathway, which inhibits CeAm output neurons through feed-forward GABAergic inhibition, is anxiolytic, reducing anxiety behavior in mice and rats (Tye et al., 2011; Cai et al., 2018; Zhou et al., 2021). Previous pain research has provided ample evidence for the involvement of the BLA–CeAl and PBN–CeAl pathways in pain. Excitatory synaptic activity in CeA has been shown to be increased in pain conditions (Ren and Neugebauer, 2010; Zhou et al., 2021). Optical activation of the anxiolytic BLA-CeA pathway inhibits pain behavior (Cai et al., 2018; Zhou et al., 2021), but increases responses of spinal dorsal horn neurons and behavior of arthritis pain (Mazzitelli et al., 2021). Excitatory activity of the PBN–CeA pathway is also potentiated in pain conditions (Ikeda et al., 2007). Interestingly, this PBN–CeA pathway carries the affective aspect of pain (Han et al., 2015) and its activation is sufficient to induce behaviors of negative emotion (Cai et al., 2018).

Increasing evidence indicates that there are functionally distinct types of cells in CeA that mediate differential effects on pain (Wilson et al., 2019; Li and Sheets, 2020; Neugebauer et al., 2020; Miller Neilan et al., 2021). These previous studies primarily focus on CeA cells that express different neurochemical profiles of somatostatin (SOM), protein kinase C-delta (PKCδ) or corticotropin-releasing hormone (CRF) in regulation of pain. However, our understanding of the local amygdala circuits and synaptic connections of different cell types, beyond the main framework of the BLA–CeAl–CeAm circuit, for regulation of emotion as well as pain is still quite limited. Our recent study has shown that optical activation of the glutamate inputs of the PBN–CeAl pathway and the BLA–CeAl pathway produces opposing effects on behaviors of emotion and pain, indicating functionally opposing CeAl cells and glutamate circuits (Cai et al., 2018). In this study, we investigated the input-specific synaptic networks of CeA neurons and their synaptic plasticity in response to anxiety-associated pain in rats.

## Materials and methods

### Animals

Male and female Wistar rats weighing 150–300 g were used in this study. All procedures involving the use of animals are conducted in accordance with the guidelines by the Animal Care and Use Committee of the University of Texas MD Anderson Cancer Center. The rats were housed in a 12 h light/dark cycle with food and water readily available. To induce inflammatory pain that typically lasts 1–2 weeks (Hou et al., 2015; Cai et al., 2018), complete Freund’s adjuvant (CFA, 50 μl) was injected into the plantar surface of one hind paw of a rat under brief halothane anesthesia. Saline was similarly injected in control group of rats. All behavioral tests and recording experiments were conducted 3 days after the CFA or saline injection. Thereby, the 3-day pain condition was defined as persistent pain.

### Test of mechanical pain

Thresholds of mechanical pain were measured by the von Frey test in rats 3 days after the CFA injection. A freely moving, unrestrained rat was placed in a plastic box with mesh floor and allowed to acclimate for 20 min. A series of calibrated von Frey filaments were applied perpendicularly to the plantar surface of a hind paw with sufficient force to bend the filament. A brisk movement of the hind paw (withdrawal or flinching) was considered as a positive response. The threshold (g) of the tactile stimulus producing a 50% likelihood of withdrawal was determined by the “up-down”-calculating method (Chaplan et al., 1994). The paw-withdrawal response was measured twice with a 5-min interval.

### Test of anxiety

The open field test was used to test anxiety behavior in rats (Fernando and Robbins, 2011). It was conducted in an illuminated chamber (72 × 72 × 30 cm) including a central zone and an outer zone. A rat was placed in the center of the chamber and allowed to move freely for 15 min. Locomotor activity of the rat was video-recorded and analyzed with an automated video-tracking system (EthoVision XT, Noldus Information Technology). Reduced time the rat spent and reduced distance the rat traveled in the central zone was regarded as an index of anxiety behavior. Total distance the rat traveled in the entire chamber during the test was also analyzed to evaluate general locomotor activity of the rat.

### Adeno-associated viral vectors and viral injection

The adeno-associated viral (AAV) vector serotype 5, AAV5-CaMKIIα-hChR2 (H134R)-mCherry, was obtained from the Vector Core Facility at The University of North Carolina at Chapel Hill. The vector was bilaterally microinjected (1 μl each side) into PBN (anteroposterior, –7.5 to –8.5 mm from the bregma; lateral, ± 1.9–2.0 mm; ventral, –6.1 to 6.2 mm from dura) in rats under anesthesia in a stereotaxic apparatus. Experiments were performed at least 4 weeks after the vector injection. After the experiments, brain tissues were harvested for anatomical verification of the injection sites within PBN.

### Immunohistochemistry

A rat was deeply anesthetized with pentobarbital and transcardially perfused with heparinized saline and subsequently with ice-cold 4% paraformaldehyde in 1 × PBS (pH 7.4). The brain was removed and post-fixed in 4% paraformaldehyde overnight at 4°C, followed by dehydration with 30% sucrose in 1 × PBS. Tissues were sectioned into 30-μm thick coronal sections with a cryostat at –20°C. Sections were blocked with 5% normal donkey serum in PBS containing 0.3% Triton X-100 and incubated overnight with primary antibodies: mouse or rabbit anti-mCherry antibody, 1:500 dilution (Abcam Cat# ab167453, RRID:AB_2571870). Sections were then rinsed and incubated with the goat anti-mouse or goat anti-rabbit Alexa Fluor-conjugated secondary antibody, 1:500 (Molecular Probes Cat# A-11008, RRID:AB_143165 or Cat# A-11004, RRID:AB_141371). The sections were then mounted on slides, dried and coverslipped with ProLong Gold anti-fade reagent. The stained sections were examined with an Olympus BX51 fluorescence microscope or a Zeiss 710 confocal microscope.

### Whole-cell recording

Brain slices (250 μm thick) containing the amygdala were obtained from CFA- or saline-injected rats and cut in a vibratome in the ice-cold dissection buffer (in mM): 126 NaCl, 2.5 KCl, 1.2 NaH_2_PO_4_, 3.6 MgCl_2_, 11 D-Sucrose, and 25 NaHCO_3_, saturated with 95% O_2_ and 5% CO_2_, pH 7.2–7.4. For recording, a slice was placed in a recording chamber and perfused with preheated (35°C) physiological saline (external solution) containing the following (in mM): 126 NaCl, 2.5 KCl, 1.2 NaH_2_PO_4_, 1.2 MgCl_2_, 2.4 CaCl_2_, 11 glucose, and 25 NaHCO_3_, saturated with 95% O_2_ and 5% CO_2_, pH 7.2–7.4. Whole-cell voltage-clamp recording was obtained with a glass pipette (resistance 2–5 MΩ) filled with an internal solution containing the following (in mM): 126 KCl, 10 NaCl, 1 MgCl_2_, 11 EGTA, 10 HEPES, 2 ATP, and 0.25 GTP, pH adjusted to 7.3 with KOH; osmolarity, 280–290 mOsM. The high concentration of chloride in the recording pipette makes the GABAergic inhibitory postsynaptic current (IPSC) in a downward direction with a holding potential of –70 mV (Zhang and Pan, 2010). Whole-cell recordings were conducted with Multiclamp 700B patch-clamp amplifier (Molecular Devices) and data were acquired with a Digidata 1550B acquisition system (Molecular Devices) digitized at 10 kHz and low pass filtered at 2 kHz. CeAl and CeAm in CeA slices were visually identified under the microscope by landmarks and our immunohistochemical images.

Recordings of excitatory postsynaptic currents (EPSCs) were made in the presence of the GABA_*A*_ receptor antagonist picrotoxin (50 μM). IPSC recordings were made in the NMDA receptor antagonist D-AP5 (50 μM) and the non-NMDA glutamate receptor antagonist CNQX (10 μM). Miniature EPSCs or miniature IPSCs were recorded in tetrodotoxin (1 μM). Electrical stimuli of constant currents (0.25 ms, 200–600 μA) were used to evoke postsynaptic currents with a bipolar stimulating electrode placed in BLA or in CeAl. Paired-pulse ratio (PPR) was obtained by a pair of electrical stimuli (400 μA, 100 ms interval) and was calculated by dividing the amplitude of the second postsynaptic current by the first one (Zhang et al., 2011). To calculate the AMPA/NMDA ratio, KCl was replaced by CsCl, and QX-314 (1 mM) and TEA-Cl (5 mM) were included in the internal solution. A total EPSC was first recorded and then the AMPA EPSC was recorded at + 40 mV in the presence of D-AP5 (50 μM). The NMDA EPSC was determined by subtracting the AMPA EPSC from the total EPSC (Cai et al., 2013). For recording of quantal IPSCs (qIPSCs), Ca^2+^ was replaced by Sr^2+^ in the external solution in the presence of D-AP5 (50 μM) and CNQX (10 μM). qIPSCs were analyzed during a 300 ms period beginning 50 ms after each electrical stimulus (Wan et al., 2014). To evoke a light-induced EPSC, a 473 nm DPSS laser (Shanghai Laser and Optic Century Co., China) was used to deliver blue light with 5 ms duration and 10 mW power controlled by a Master-8 pulse stimulator (A.M.P.I) through an optical fiber to a cell being recorded in the slice. Due to significant variations in the amplitude of EPSCs at low light intensities, a single near-maximum light intensity (10 mW) was used to evoke EPSCs for between-group comparisons. All data were analyzed by Clampfit 10.7 and Igor pro 6 (WaveMetrics).

### Statistical analysis

Group data were statistically tested with the non-paired or paired, two-tailed Student’s *t*-test, one-way ANOVA, or two-way ANOVA with the Bonferroni *post hoc* test for repeated measures of multiple comparisons as appropriate. All statistical tests were performed with Prism 9 software. A *p*-value < 0.05 was considered statistically significant. The data were presented as means ± SEM.

## Results

### Rat model of persistent pain and anxiety

**FIGURE 1:**
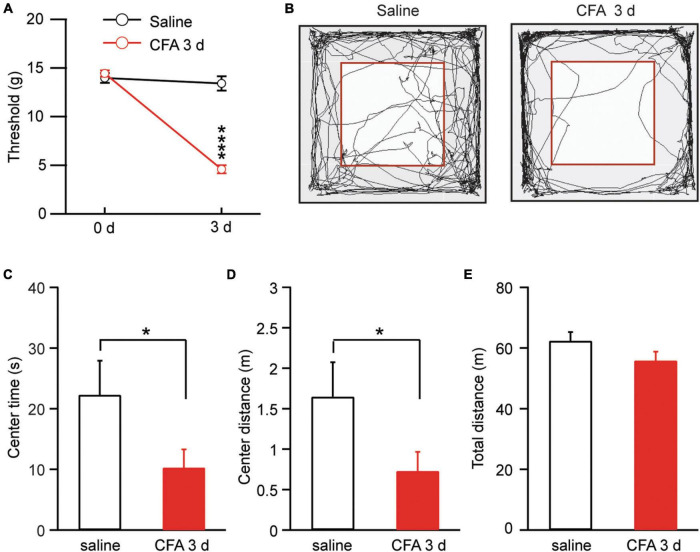
Persistent pain induces anxiety-like behavior. **(A)** Thresholds of mechanical pain in rats 3 days after an intra-paw injection of saline (*n* = 15) or complete Freund’s adjuvant (CFA, 50 μl, *n* = 17). **(B)** Locomotion traces of a rat injected with saline or CFA in an open field test. The squares inside the chamber mark the central zone. **(C–E)** Group data of the time the rats spent in the central zone **(C)**, and distance the rats traveled in the central zone **(D)** and in the entire test chamber **(E)** during the test for the saline and CFA groups of rats. *p < 0.05 and ****p ≤ 0.0001.

We used complete Freund’s adjuvant (CFA, 50 μl) to induce lasting inflammatory pain in rats. Three days after an intra-paw injection of CFA, rats displayed significantly sensitized pain, as measured by the von Frey test for mechanical pain of allodynia, when compared with saline-injected control rats [saline: 0 day, 13.98 ± 0.51, 3 day, 13.40 ± 0.73, *n* = 15; CFA: 0 day, 14.44 ± 0.33, 3 day, 4.61 ± 0.41, *n* = 17. Day, *F*_(1, 30)_ = 114.60, *p* < 0.0001; CFA: *F*_(1, 30)_ = 64.47, *p* < 0.0001; two-way ANOVA with the Bonferroni test; Figure 1A]. This pain condition for 3 days was defined as persistent pain (Hou et al., 2015; Cai et al., 2018). We then determined whether this persistent pain would induce changes in animal behavior of anxiety. Rats with CFA-induced persistent pain were tested for anxiety behavior by the open field test. Our results showed that, 3 days after the CFA injection, rats displayed significant anxiety-like behavior when compared to saline-injected rats, spending significant less time and traveling less distance in the central zone (central time: saline, 22.3 ± 5.6 s, *n* = 17; CFA, 10.3 ± 3.0 s, *n* = 15, *p* = 0.03; central distance: saline, 1.65 ± 0.43 m, *n* = 17; CFA, 0.73 ± 0.24 m, *n* = 15, *p* = 0.03, unpaired *t*-test; Figures 1B–D). There was no difference in the total distance the rats traveled during the test between the two groups, indicating that the locomotor activity of the rats was not affected by the pain condition (saline, 62.37 ± 2.88 m, *n* = 17; CFA, 55.88 ± 2.89 m, *n* = 15, *p* = 0.14, unpaired *t*-test; Figure 1E). Thus, it seems that the persistent pain can induce anxiety behavior as measured by the open field test in rats.

### Persistent pain activates basolateral amygdala–lateral division of the central nucleus of the amygdala–medial division of the central nucleus of the amygdala circuit in amygdala

**FIGURE 2:**
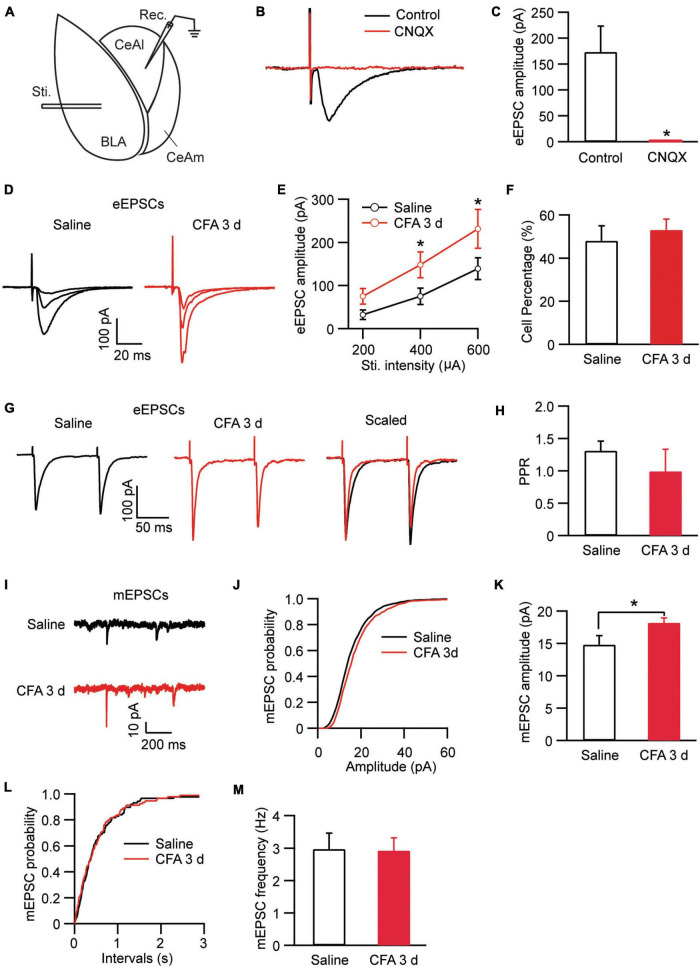
Persistent pain activates the BLA–CeAl pathway. **(A)** A schematic of recording configuration in amygdala with electrical stimulation (Sti.) in BLA and whole-cell recording (Rec.) in CeAl. **(B,C)** Sample traces **(B)** and group data **(C)** of evoked EPSCs (eEPSCs) in CeAl neurons before (control) and after application of CNQX (10 μM), *n* = 7. **(D,E)** Representative traces **(D)** and group data **(E)** of eEPSCs evoked by various stimulation intensities in the saline- and CFA-injected rats (*n* = 9–16 per group). **(F)** Percentage of CeAl cells displaying BLA-elicited eEPSCs in the saline and CFA groups. **(G,H)** Current traces **(G)** and group data **(H)** of eEPSC pairs evoked by two electrical stimuli (400 μA, 100 ms interval) in the two rat groups. **(I–M)** Representative traces of miniature EPSCs (mEPSCs, **I**), group data of mEPSC amplitude **(J,K)** and mEPSC frequency **(L,M)** in the saline and CFA groups. *p < 0.05.

In amygdala slices from CFA- or saline-injected rats, we made whole-cell recordings from neurons in CeAl and recorded electrically evoked excitatory postsynaptic current (eEPSC) from synaptic inputs of BLA neurons by electrical stimulation through a bipolar stimulating electrode placed within BLA (Figure 2A). Our results showed that the eEPSC of BLA–CeAl pathway was completely blocked by CNQX (10 μM) (control, 173.3 ± 50.94, CNQX, 1.34 ± 0.75, *n* = 7, *p* = 0.016, paired *t*-test; Figures 2B,C), confirming that the eEPSC is mainly mediated by glutamate AMPA receptors (AMPARs) in this recording condition (Cai et al., 2013). In CFA-injected rats, we found that persistent pain dramatically increased the amplitude of the BLA–CeAl eEPSC with various stimulus intensities when compared to saline-injected control rats [stimulus intensity: *F*_(2, 38)_ = 14.90, *p* < 0.0001; CFA: *F*_(1, 28)_ = 6.63, *p* = 0.0156, two-way ANOVA with the Bonferroni test; Figures 2D,E]. No difference was found for the percentage of CeAl cells receiving the projection from BLA between the saline and pain groups (saline, 48% ± 7%, *n* = 12, CFA, 53% ± 5%, *n* = 11, *p* = 0.29, unpaired *t*-test; Figure 2F). The paired pulse ratio (PPR), which is inversely related to the release probability of presynaptic neurotransmitter vesicle (Zucker and Regehr, 2002), was not significantly changed by the pain condition (saline, 1.31 ± 0.15, CFA, 0.99 ± 0.34, *n* = 26, *p* = 0.07, unpaired *t*-test; Figures 2G,H), indicating that the increased eEPSC amplitude is mediated likely by postsynaptic changes. To confirm that, we recorded and analyzed the miniature EPSC (mEPSC) in the CeAl neurons. We found that the mEPSC amplitude was significantly increased in the pain group while the mEPSC frequency was similar in the saline and pain groups (amplitude: saline, 14.7 ± 1.4 pA, *n* = 12, CFA, 18.1 ± 0.7 pA, *n* = 10, *p* = 0.03; frequency: saline, 2.97 ± 0.4, *n* = 12, CFA, 2.92 ± 0.3, *n* = 10, *p* = 0.46, unpaired *t*-test; Figures 2I–M). These results suggest that persistent pain increases the activity of BLA–CeAl synaptic pathway predominantly by postsynaptic changes in CeAl neurons. Analysis of AMPA/NMDA ratio of the eEPSCs in these cells further supported the postsynaptic potentiation in this BLA-CeAl pathway (see results below).

**FIGURE 3:**
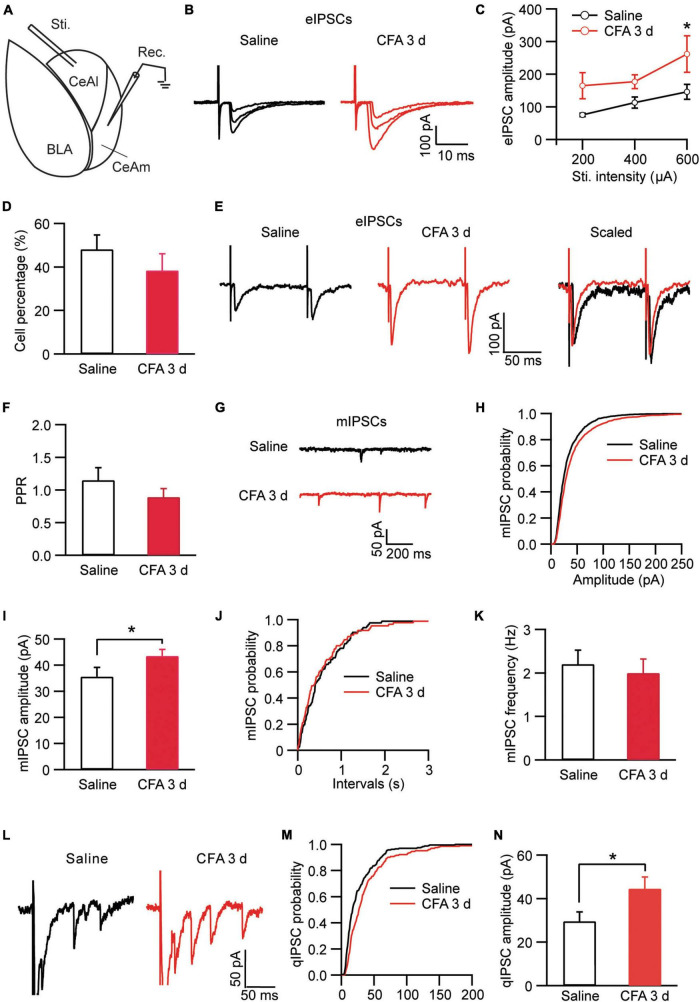
Persistent pain activates the CeAl–CeAm pathway. **(A)** A schematic of recording configuration in amygdala with electrical stimulation in CeAl and whole-cell recording in CeAm. **(B,C)** Current traces **(B)** and group data **(C)** of evoked IPSCs (eIPSCs) in CeAm neurons with various stimulation intensities in the saline and CFA groups (*n* = 4–25 per group). **(D)** Percentage of CeAm cells displaying CeAl-elicited eIPSCs in the two rat groups. **(E**,**F)** Current traces of eIPSC pairs **(E)** and group data of paired-pulse ratio (PPR, **F**) in the two rat groups. **(G–K)** Representative traces of miniature IPSCs (mIPSCs, **G**), group data of mIPSC amplitude **(H,I)** and mIPSC frequency **(J,K)** in the saline and CFA groups. **(L)** Representative current traces of an evoked IPSC (truncated) followed by quantal IPSCs (qIPSCs) from a rat in the saline and CFA groups. **(M,N)** Cumulative probability **(M)** and group data **(N)** of qIPSC amplitude in the saline and CFA groups of rats. *p < 0.05.

Neurons in CeA including both CeAl and CeAm are predominantly GABAergic and a well characterized local circuit within CeA for primary information flow is the synaptic projection from CeAl to CeAm (Tye et al., 2011; Neugebauer et al., 2020). Next, we examined changes in this CeAl–CeAm GABAergic projection under the persistent pain condition by stimulating CeAl neurons and recording CeAm neurons in brain slices (Figure 3A). We found that CFA-induced persistent pain significantly augmented the amplitude of the evoked inhibitory postsynaptic current (eIPSC) with various stimulus intensities when compared to the saline control group [stimulus Intensity: *F*_(2, 33)_ = 4.003, *p* = 0.0278; CFA: *F*_(1, 39)_ = 7.392, *p* = 0.0097, two-way ANOVA with the Bonferroni test; Figures 3B,C]. There was no difference in the percentage of CeAm cells with eIPSCs between the pain and control groups (saline, 48.1% ± 6.6%, *n* = 7, CFA, 38.4% ± 7.7%, *n* = 7, *p* = 0.18, unpaired *t*-test; Figure 3D). The pain condition did not have a significant effect on the PPR of those eIPSCs [saline, 1.14 ± 0.19, *n* = 22, CFA, 0.88 ± 0.13, *n* = 16, *p* = 0.15, unpaired *t*-test; Figures 3E,F). Further analysis of miniature IPSCs (mIPSCs) revealed that the pain condition significantly enhanced the amplitude, but not the frequency, of the mIPSCs in CeAm neurons (amplitude: saline, 35.6 ± 3.5 pA, *n* = 12, CFA, 43.5 ± 2.5 pA, *n* = 14, *p* = 0.03; frequency: saline, 2.2 ± 0.3, *n* = 12, CFA, 2.0 ± 0.3, *n* = 14, *p* = 0.36, unpaired *t*-test; Figures 3G–K), suggesting a postsynaptic mechanism. To determine whether this postsynaptic potentiation indeed occurred in the CeAl-CeAm pathway, we analyzed quantal IPSCs (qIPSCs) obtained by evoking IPSCs with Ca^++^ in the external solution replaced by Sr^++^, which induces asynchronous quantal events after the initial synchronous release (Wan et al., 2014). We found that persistent pain also significantly increased the amplitude of qIPSCs when compared to the control group (saline: 29.5 ± 3.6 pA, *n* = 8; CFA: 44.5 ± 5.4 pA, *n* = 9, *p* = 0.04, unpaired *t*-test; Figures 3L–N). These results suggest that the pain condition enhances the activity of CeAl–CeAm GABAergic projections predominantly by a postsynaptic mechanism in CeAm neurons. Thus, it appears that, under the persistent pain condition, activity of the amygdala BLA–CeAl–CeAm circuit, including the BLA–CeAl glutamatergic pathway and the CeAl–CeAm GABAergic pathway, is significantly activated, suggesting an increased feed-forward inhibition of CeAm neurons of the amygdala output under the pain condition.

### Synaptic inputs define distinct cell types within amygdala circuits

**FIGURE 4:**
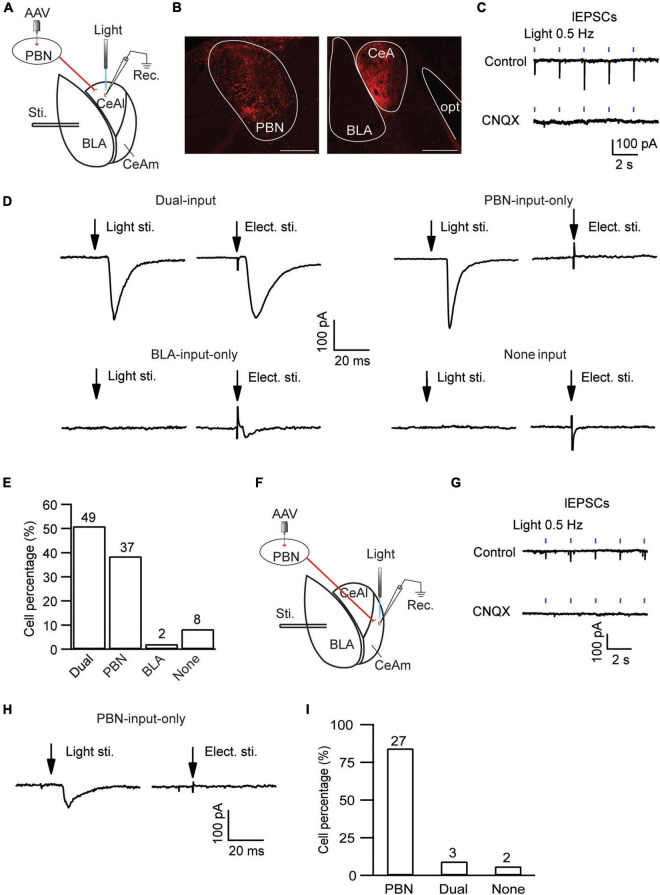
Distinct, input-specific types of cells in CeAl and CeAm from naïve rats. **(A)** A schematic of experimental settings for cell types in CeAl, with the adeno-associated viral (AAV) vector, AAV-CaMKII-hChR2-mCherry, injected in the PBN and 4 weeks later, electrical stimulation in BLA, and light (optical) stimulation and whole-cell recording in CeAl. **(B)** Images of fluorescence staining in transfected cells in PBN (Left) and projection terminals in amygdala (Right). Scale bars = 500 μm. opt, optic tract. **(C)** Sample traces of light-evoked EPSCs (lEPSCs) by 473 nm light at 0.5 Hz in CeAl neurons before (control) and after application of CNQX (10 μM), *n* = 4. **(D)** Representative EPSC traces in CeAl cells that display (1) both PBN-elicited lEPSC and BLA-elicited eEPSC (dual-input), (2) lEPSC only (PBN-input only), (3) eEPSC only (BLA-input only), and (4) no EPSC (none input). eEPSCs were evoked by 600 μA. **(E)** Number and percentage of each cell type in the population of CeAl cells recorded. **(F)** A schematic of experimental settings similar to A, but with light stimulation and whole-cell recording in CeAm. **(G)** Traces of lEPSCs in CeAm neurons before (control) and after application of CNQX (10 μM), *n* = 4, with the same light stimulation as in C. **(H)** Current traces in a CeAm cell with lEPSC only (PBN-input only). **(I)** Percentage of three cell types found in CeAm. The numbers on top of each column indicate the cell number in each group of the cell types.

CeAl has been shown to function as a main processing center of the amygdala in regulation of emotion as well as pain, receiving direct synaptic inputs both from BLA and from PBN (Hunt and Mantyh, 2001; Han and Neugebauer, 2004; Ikeda et al., 2007; Ren et al., 2013; Han et al., 2015; Neugebauer, 2015; Cai et al., 2018). We then examined the local circuit of CeAl neurons and their differential synaptic inputs from BLA and PBN in naïve rats. To identify CeAl inputs from PBN, we injected the AAV5-CHR2-mCherry vector into the PBN of naïve rats to transfect PBN neurons and their glutamatergic projections to CeAl, and 4 weeks after the injection, a blue light (474 nm) was delivered to a CeAl neuron under whole-cell recording in an amygdala slice to evoke a light-induced EPSC (lEPSC) from the transfected PBN–CeAl pathway (Figure 4A). Figure 4B shows the fluorescence signals in the injected PBN and exclusively in CeA (mostly CeAl) of the same rat. In CeAl neurons under whole-cell recording, a light stimulation evoked an EPSC in a repeated and time-matched fashion, and the lEPSCs were completely blocked by CNQX (10 μM), suggesting that the lEPSC of the PBN–CeAl pathway was mediated mostly by AMPARs (Figure 4C). Combining this PBN-elicited lEPSC and the eEPSC from BLA, we analyzed CeAl neurons and their synaptic inputs to understand the local cellular circuits. Based on the synaptic inputs, we found a total of 4 types of CeAl neurons with distinct input profiles (Figures 4D,E): 1) Dual-input cells, which consisted of the majority of CeAl cells recorded (49 of 96 cells, 51%), displayed both PBN-elicited lEPSCs and BLA-elicited eEPSCs; 2) PBN-input-only cells, the second abundant type (37 of 96 cells, 39%), had only PBN-elicited lEPSC, but no BLA-elicited eEPSC under the same stimulation conditions; 3) BLA-input-only cells (2 of 96, 2%) displayed only a small BLA-elicited eEPSC; and 4) none-input cells (8 of 96, 8%) had neither the lEPSC nor the eEPSC under the same conditions. These results demonstrate that the largest population of CeAl neurons (51%) receives glutamatergic synaptic inputs from both PBN and BLA whereas the second abundant CeAl neurons (39%) receive glutamatergic synaptic inputs only from PBN.

Next, we examined the synaptic input profiles of neurons in CeAm, using similar optogenetic strategy and whole-cell recording in naïve rats with AAV5-CHR2-mCherry injected in the PBN (Figure 4F). Similar lEPSCs were repeatedly evoked by light stimuli in CeAm neurons and were also completely blocked by CNQX (10 μM), suggesting that PBN also has direct glutamatergic projections to CeAm neurons (Figure 4G). Using similar analytic methods of input profiles, we found that the majority of CeAm cells (27 of 32 cells, 84.3%) were the type of PBN-input-only cells, receiving synaptic inputs only from PBN, but not BLA (Figures 4H,I).

**FIGURE 5:**
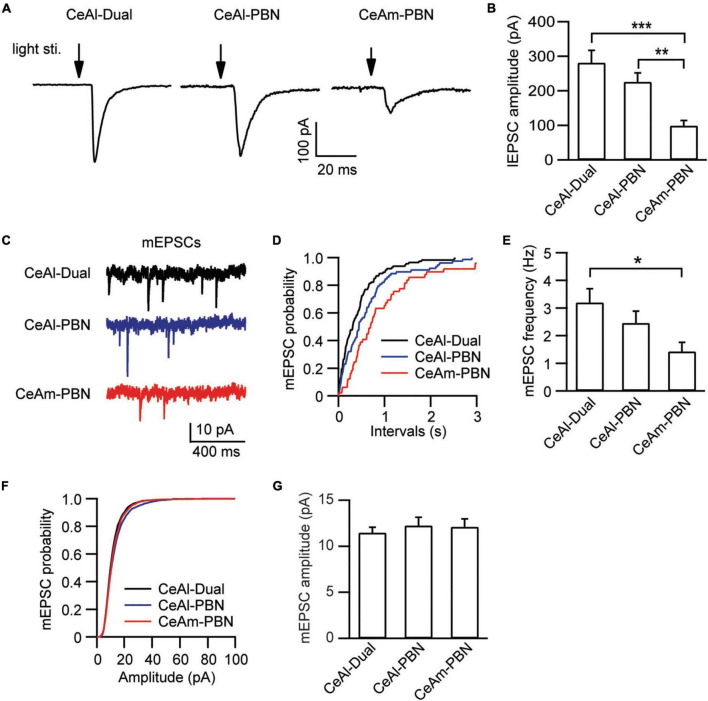
Synaptic strength is stronger in PBN–CeAl synapses than in PBN–CeAm synapses in naïve rats. **(A,B)** lEPSC traces **(A)** and group data of lEPSC amplitude **(B)** in dual-input cells and PBN-input-only cells in CeAl, and in PBN-input-only cells in CeAm. **(C–G)** mEPSC traces **(C)** and group data of mEPSC frequency **(D,E)** and mEPSC amplitude **(F,G)** in the 3 cell types in CeAl and CeAm. *p < 0.05, **p < 0.01, and ***p < 0.001.

In these different types of CeAl and CeAm neurons under the same stimulation conditions in naïve rats, we also analyzed the amplitude of their EPSCs, which may indicate relative synaptic strength among these cell types. Our results showed that the PBN-elicited lEPSC amplitudes were similar in the two major cell types in CeAl (dual-input, 281.4 ± 36.2 pA, *n* = 51, PBN-input-only, 225.7 ± 25.9 pA, *n* = 49, *p* = 0.17), but they were significantly larger than the PBN-elicited lEPSC in CeAm cells (PBN-input-only in CeAm, 99.0 ± 15.4 pA, *n* = 29; vs. dual-input: *p* = 0.0002, vs. PBN-input-only in CeAl: *p* = 0.008, one-way ANOVA with multiple comparisons; Figures 5A,B). For the BLA-elicited eEPSC in CeAl cells, it was overwhelmingly larger in dual-input cells than in the few BLA-input-only cells (dual-input, 161.1 ± 31.6 pA, *n* = 58; BLA-input-only, 32 pA on average of 2 cells, evoked by 600 μA stimulation, Figure 4D). We further analyzed the mEPSCs in these cell types to evaluate their intrinsic strength of synaptic activities in naive conditions. We found that the mEPSC frequencies in the two major cell types in CeAl were not significantly different (dual-input, 3.2 ± 0.5, PBN-input-only, 2.4 ± 0.4, *n* = 15, *p* = 0.26), but it was significantly higher in dual-input cells than the mEPSC frequency in CeAm cells of PBN-input-only (1.4 ± 0.3, *n* = 11, vs. dual-input: *p* = 0.01; vs. PBN-input-only in CeAl: *p* = 0.15, one-way ANOVA with multiple comparisons; Figures 5C–E). There was no difference in the mEPSC amplitude among the three cell types (dual-input CeAl, 11.4 ± 0.6 pA, *n* = 15, PBN-input-only in CeAl, 12.2 ± 0.9 pA, *n* = 15, *p* = 0.48 vs. dual-input CeAl; PBN-input-only in CeAm, 12.1 ± 0.8 pA, *n* = 11, *p* = 0.58 vs. dual-input CeAl, *p* = 0.92 vs. PBN-input-only in CeAl, one-way ANOVA with multiple comparisons; Figures 5F,G). Thus, while the recorded mEPSC may not specifically reflect the synaptic activity of the PBN inputs, these data indicate that it is likely that PBN projects more predominantly to CeAl with higher synaptic activity than its projections to CeAm. Our data of eEPSCs indicate that BLA neurons almost exclusively project to CeAl cells that also receive inputs from PBN.

### Persistent pain targets selective synaptic inputs in different types of central nucleus of the amygdala cells

**FIGURE 6:**
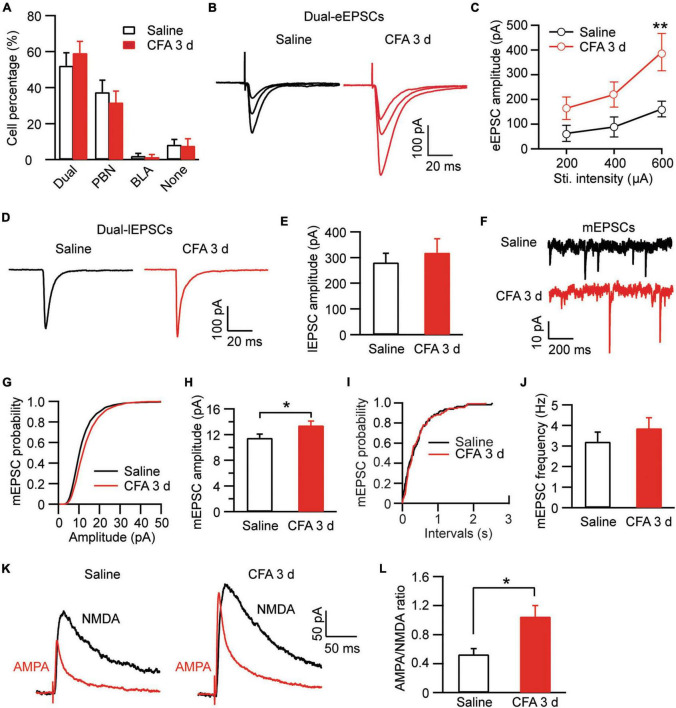
Persistent pain activates BLA–CeAl inputs, not PBN–CeAl inputs in dual-input CeAl cells. **(A)** Group data of cell percentages for dual-input cells, PBN-input-only cells, BLA-input-only cells and none-input cells in CeAl from rats injected with saline or CFA. **(B,C)** BLA-elicited eEPSC traces **(B)** and group data of the eEPSC amplitude **(C)** in the dual-input CeAl cells from the saline- and CFA-injected rats. **(D,E)** PBN-elicited lEPSC traces **(D)** and group data of the lEPSC amplitude **(E)** in the dual-input CeAl cells from the saline- and CFA-injected rats. **(F–J)** mEPSC traces **(F)** and group data of mEPSC amplitude **(G,H)** and mEPSC frequency **(I,J)** in the dual-input CeAl cells from the saline and CFA rat groups. **(K,L)** Representative current traces of AMPA EPSCs and NMDA EPSCs **(K)**, and group data of AMPA/NMDA ratio **(L)** in the saline and CFA groups. *p < 0.05 and **p < 0.01.

After identifying different cell types and their baseline synaptic activities in amygdala, we were wondering how their activities would be changed by the CFA-induced aversive conditions of persistent pain and anxiety. Firstly, we examined the percentage of cell types in the cell population of CeAl. There was no significant difference in the percentage of each of the 4 cell types in CeAl between the saline and CFA groups [Dual-input: saline, 52.2% ± 7.1%, *n* = 13, CFA, 59.2% ± 6.5%, *n* = 9, *p* > 0.99; PBN-input-only: saline, 37.5% ± 6.7%, *n* = 13, CFA, 31.7% ± 6.4%, *n* = 9, *p* > 0.99; BLA-input only: saline, 2.1% ± 1.4%, *n* = 13, CFA, 1.4% ± 1.4%, *n* = 9, *p* > 0.99; none-input: Saline, 8.2% ± 2.9%, *n* = 13, CFA, 7.7% ± 3.9%, *n* = 9, *p* > 0.99; CFA: *F*_(1, 32)_ < 0.0001, *p* > 0.99, cell type: *F*_(3, 48)_ = 31.18, *p* < 0.0001, two-way ANOVA with the Bonferroni test; Figure 6A]. This indicates that persistent pain does not change the profiles of synaptic inputs in CeAl neurons. However, in dual-input cells, we found that persistent pain significantly increased the amplitude of BLA-elicited eEPSCs and thus the exciility of CeAl cells [stimulus intensity: *F*_(2, 37)_ = 4.253, *p* = 0.0218; CFA: *F*_(1, 89)_ = 4.012, *p* = 0.0482, two-way ANOVA with the Bonferroni test; Figures 6B,C]. In contrast, the amplitude of PBN-elicited lEPSCs in these dual-input cells remained unchanged between the control and CFA groups (saline, 281.3 ± 36.1 pA, *n* = 51, CFA, 319.2 ± 54.2 pA, *n* = 40, *p* = 0.27, unpaired *t*-test; Figures 6D,E). Our analysis of mEPSCs in the dual-input cells revealed that persistent pain significantly increased the mEPSC amplitude without affecting the frequency (amplitude: Saline, 11.4 ± 0.6 pA, *n* = 15, CFA, 13.4 ± 0.6 pA, *n* = 15, *p* = 0.02; frequency: saline, 3.2 ± 0.5, *n* = 15, CFA, 3.8 ± 0.5, *n* = 15, *p* = 0.11, unpaired *t*-test; Figures 6F–J], indicating a postsynaptic mechanism involved in the pain-enhanced eEPSCs. To further verify that the synaptic potentiation of eEPSCs occurred in the specific BLA-CeAl pathway of these cells, we examined the AMPA/NMDA ratio of the BLA-elicited eEPSCs. Our results showed that the AMPA/NMDA ratio was significantly increased by persistent pain in these dual-input cells (saline: 0.53 ± 0.08, *n* = 6; CFA, 1.05 ± 0.15, *n* = 9, *p* = 0.02, unpaired *t*-test; Figures 6K,L). These results suggest that, in dual-input cells in CeAl, persistent pain with associated anxiety selectively enhances synaptic strength and intrinsic synaptic activity of direct glutamatergic inputs from BLA through a postsynaptic mechanism whereas it does not change the synaptic activity of glutamatergic inputs from PBN in the same cells.

**FIGURE 7:**
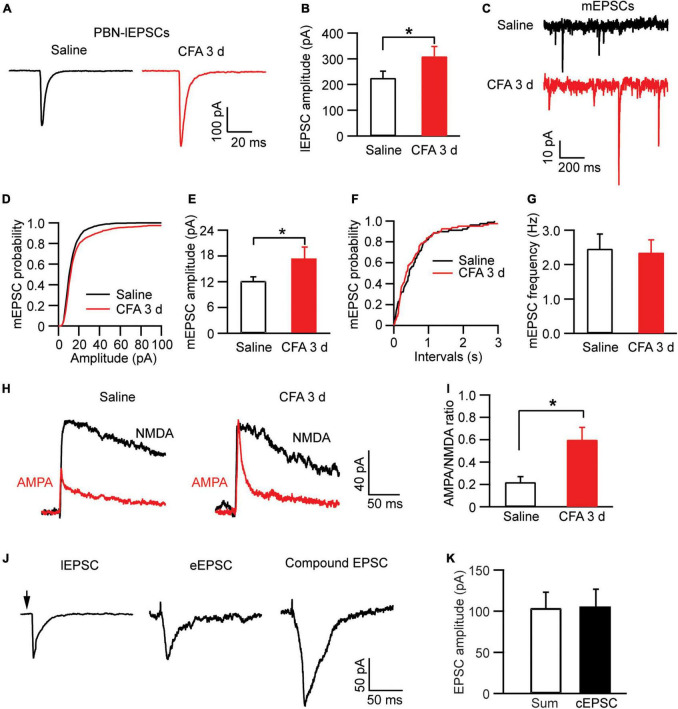
Persistent pain activates PBN–CeAl inputs in PBN-input-only CeAl cells. **(A,B)** PBN-elicited lEPSC traces **(A)** and group data of lEPSC amplitude **(B)** in the PBN-input-only cells in CeAl from the saline and CFA rat groups. **(C–G)** mEPSC traces **(C)** and group data of mEPSC amplitude **(D,E)** and mEPSC frequency **(F,G)** in the PBN-input-only cells in CeAl from the saline and CFA rat groups. **(H,I)** Representative current traces of AMPA EPSCs and NMDA EPSCs **(H)**, and group data of AMPA/NMDA ratio in the saline and CFA groups. **(J)** Representative current traces of a lEPSC and an eEPSC, and a compound EPSC (cEPSC) evoked by simultaneous light and electrical stimulation in the same dual-input cell. **(K)** Group data of amplitudes of the sum of the lEPSC and eEPSC, and cEPSC in each dual-input cell. *p < 0.05.

We then examined the pain effect on synaptic inputs in PBN-input-only cells in CeAl. We were excited to find that, in strong contrast to dual-input cells, the amplitude of lEPSCs evoked by the same light intensity was significantly augmented in the pain condition when compared to controls (saline, 225.7 ± 25.9 pA, *n* = 49, CFA, 310.1 ± 38.2 pA, *n* = 38, *p* = 0.03, unpaired *t*-test; Figures 7A,B). Furthermore, we found that, in these PBN-input-only cells in CeAl, the amplitude of mEPSCs was significantly increased in the pain group without apparent changes in mEPSC frequency (amplitude: saline, 12.2 ± 0.9 pA, *n* = 15, CFA, 17.2 ± 2.8 pA, *n* = 15, *p* = 0.04; frequency: saline, 2.4 ± 0.4, *n* = 15, CFA, 2.3 ± 0.3, *n* = 15, *p* = 0.42, unpaired *t*-test; Figures 7C–G), suggesting again a dominant postsynaptic mechanism for the increased synaptic strength of the solely PBN inputs in this type of CeAl cells under the pain condition. To verify that the postsynaptic enhancement of lEPSCs was specifically associated with the PBN–CeAl pathway, we similarly analyzed the AMPA/NMDA ratio of the PBN-elicited lEPSCs. We found that persistent pain also significantly increased the AMPA/NMDA ratio in these PBN-input-only cells (saline: 0.21 ± 0.05, *n* = 6; CFA, 0.60 ± 0.11, *n* = 6, *p* = 0.01, unpaired *t*-test; Figures 7H,I), confirming a postsynaptic mechanism for the synaptic potentiation of the PBN–CeAl pathway. Due to the rare and a very small number of BLA-input-only cells found in CeAl, we did not intend to analyze the difference in the small amplitude of eEPSCs between control and pain groups.

Finally, to determine the relative independence of EPSCs from electrically activated BLA–CeAl inputs and light-activated PBN–CeAl inputs, we recorded the eEPSCs and lEPSCs simultaneously in the same dual-input CeAl cells. To avoid a ceiling effect on the EPSC amplitude, we used below-maximum intensity in both light and electrical stimulation to evoke lEPSCs and eEPSCs. As shown in Figures 7J,K, in a dual-input cell, both light and electrical stimulation evoked an EPSC (lEPSC and eEPSC), and simultaneous light and electrical stimulation induced a compound EPSC (cEPSC) that was statistically similar to the sum of the sole lEPSC and eEPSC in amplitude (sum, 103.7 ± 19.3 pA, cEPSC, 104.7 ± 22.0 pA, *n* = 4, *p* = 0.84, paired *t*-test). These results suggest that the PBN–CeAl input and the BLA–CeAl input are largely independent in dual-input CeAl cells.

## Discussion

**FIGURE 8:**
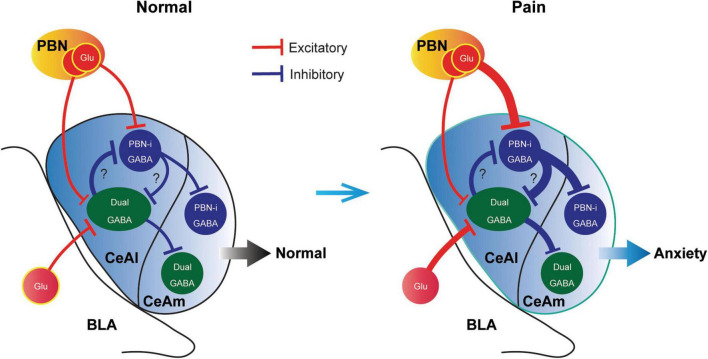
A schematic illustrating a working model of opposing cell types and synaptic circuits in normal and pain-anxiety conditions. Glu, glutamate; PBN-i, PBN-input-only.

In this study, we have identified two major types of CeAl cells with differential profiles of synaptic inputs and have revealed unique synaptic plasticity in the inputs of these two cell types under an anxiety-associated pain condition. These cell types with differential adaptive changes in their synaptic inputs may represent functionally distinct populations of CeA neurons in amygdala circuits for regulation of pain and emotion. Given our finding that pain activated the PBN inputs only to PBN-input-only cells (Figures 7A,B), but not to dual-cells in CeAl (Figures 6D,E), and that optogenetic activation of the PBN–CeAl pathway elicits negative emotion without affecting baseline pain sensitivity (Cai et al., 2018), it is thus likely that these PBN-input-only cells in CeAl exclusively encode the emotional component of peripheral pain signals and play a central role in linking pain and negative emotion in amygdala circuits. Our findings also show that the dual-input CeAl cells are the only main cell population that receive direct synaptic inputs from BLA. Since optogenetic activation of the BLA–CeAl pathway decreases negative emotion (Tye et al., 2011; Cai et al., 2018), inhibits pain and produce reward in normal conditions (Cai et al., 2018), and BLA functions as a hub for interactions between cortical structures and the amygdala (McCool et al., 2010; Etkin et al., 2011), we have proposed that this BLA–CeAl–CeAm pathway could be part of a top-down control mechanism from cortical structures that counter-balance effects of aversive stimuli and environment such as pain (Cai et al., 2018). Supporting this notion, our current findings demonstrate that pain can activate and sensitize the synaptic activity of the BLA–CeAl–CeAm circuit. Based on our current findings, we propose a working model of the amygdala circuits that regulate anxiety and pain, as shown in Figure 8. Thus, for amygdala regulation of pain and emotion, there are two functionally opposing circuits: the negative (pain, anxiogenic) PBN–CeAl–CeAm circuit where PBN-input-only cells are the major component, and the positive (analgesic, anxiolytic, reward) BLA–CeAl–CeAm circuit where the dual-inputs cells are the key component. The two opposing circuits could mutually inhibit each other through mutual GABAergic inhibition between different types of GABAergic cells as suggested in recent studies (Li et al., 2013). By our working model (Figure 8), in normal conditions, the activities of the two opposing circuits are in balance; under pain conditions, pain can activate both circuits, but through different circuits and thus distinct mechanisms with differential magnitude, time course and adaptations. Pain activates the negative circuit by direct PBN–CeAl glutamate inputs and as pain persists in persistent and chronic pain, the constant pain signal induces increasingly stronger sensitization in the negative inputs of PBN to PBN-input-only cells (not to dual-inputs cells) in CeAl. In contrast, pain activates the positive circuit indirectly, e.g., via spinothalamic pathways to cortical structures and then via corticolimbic pathways to amygdala’s BLA–CeAl inputs as a response to the pain condition. This adaptive process may be influenced by cognitive processing and overall environmental/emotional state on an individual basis, which may get impaired over the course of persistent and chronic pain development, resulting in increasingly inefficient influence of the positive circuit. Thus, the disproportional activation of the negative circuit by persistent and chronic pain overcomes the adaptive or impaired effects of the positive circuit, leading to anxiety.

Functionally different cell types and their local networks in amygdala have begun to be explored only recently for their roles in regulation of pain and emotion [for reviews, see Tye et al. (2011) and Neugebauer et al. (2020)]. Previous studies have mostly used the expression of a specific neurochemical marker for the base of cell type identification in amygdala. These markers include SOM, PKCδ, CRF, neurotensin and tachykinin (Li et al., 2013; Hunt et al., 2017; McCullough et al., 2018; Wilson et al., 2019; Li and Sheets, 2020; Miller Neilan et al., 2021). For example, PBN has been shown to project to both SOM-expressing [SOM(+)] and CRH(+) CeA cells (Li and Sheets, 2020); optical activation of PKCδ(+) or CRH(+) CeA cells increases pain response whereas optical activation of SOM(+) CeA cells decreases pain response under pain conditions (Wilson et al., 2019; Mazzitelli et al., 2021; Chen et al., 2022). Thus, it appears that PBN projects to neurochemically different cell types in CeA that have differential roles in modulation of pain. Interestingly, optical activation of SOM(+) cells in CeA induces anxiety behavior without changing pain sensitivity in normal condition (Chen et al., 2022) and drives negative emotion response of fear (Li et al., 2013). This raises the possibility that the SOM(+) cells are functionally similar to the PBN-input-only cells in inducing negative emotion. Of note is that high levels of colocalization for the mRNAs encoding some of these markers have been reported in CeAl cells (McCullough et al., 2018) and it is currently unknown whether the expression profiles of these markers would change under various pain and emotion states. In input-specific CeAl cells, we found no significant changes in the input profiles of CeA cells after 3 days of persistent pain. Our current results and working model provide a potential synaptic and circuit mechanism for roles of PBN and BLA inputs onto separate CeAl cell types in amygdala modulation of pain and associated negative emotion.

It has been well documented that general neuronal activity in amygdala is increased under various pain conditions (Neugebauer et al., 2020). Particularly, the presynaptic metabotropic glutamate receptor subtype 1 has been shown to mediate increased excitatory synaptic transmission of the BLA-CeAl pathway in a rat model of arthritic pain (Ren and Neugebauer, 2010). In CeA neurons, CFA-induced pain increases frequency and amplitude of excitatory mEPSCs (Zhou et al., 2021) and on BLA–CeA synapses, postsynaptic GluA2-containing AMPARs are involved in neuropathic pain-induced effects and comorbid negative emotion (Jiang et al., 2021). Our results of CFA-increased activity of the PBN–CeAl and BLA–CeAl–CeAm circuits are consistent with these previous studies, which may reflect an overall heightened activity state of the amygdala circuits that maintain the state of pain and negative emotion. Our analysis of mEPSCs and the AMPA/NMDA ratio of eEPSCs indicate a predominant postsynaptic mechanism associated with the PBN–CeAl and BLA–CeAl synaptic pathways, which would likely involve postsynaptic AMPARs for the heightened activity. This notion is in line with our previous study showing an increased expression of GluA1 subunits of AMPARs in CeA neurons under CFA-induced persistent pain (Hou et al., 2015). AMPARs are especially crucial for emotional events of learning and memory through activity-dependent synaptic strengthening via re-composition of GluA1 and GluA2 subunits (Malinow and Malenka, 2002; Maren, 2005; Shepherd and Huganir, 2007; Bowers et al., 2010; Clem and Huganir, 2010; Krugers et al., 2010; McCool et al., 2010; Cole et al., 2012; Cai et al., 2013, 2020). This AMPAR strengthening is achieved by switching from low conductance, GluA2-containing AMPARs to high conductance, homomeric GluA1 AMPARs (Geiger et al., 1995; Isaac et al., 2007; Kauer and Malenka, 2007; Shepherd and Huganir, 2007; Polgar et al., 2008; Bowers et al., 2010). Given the serial connection of the BLA–CeAl–CeAm circuit and its generally elevated activity in pain conditions, presynaptic mechanisms of glutamate release are also likely involved in the pain effects, perhaps just less predominant than the postsynaptic changes in AMPARs. While BLA is a major source of synaptic projections to CeA within the amygdala, potential contribution of excitatory inputs from passing fibers to the EPSCs in dual-input cells we recorded cannot be excluded in our BLA-stimulating settings.

Our working model is a simplified one that does not include other cell types and still-unknown synaptic networks in details that are involved in amygdala regulation of emotion and pain. For instance, the GABAergic intercalated cells located between BLA and CeA project to CeAm to provide feedforward inhibition on amygdala output in regulation of pain and fear (Pape and Pare, 2010; Viviani et al., 2011; Neugebauer et al., 2020). For CeAm, given that 30–40% of CeA cells express SOM (Hunt et al., 2017) and our results of about 50% of recorded CeAm cells having CeAl inputs with eIPSC (Figure 3D), it is thus likely that there are also two different cell types in CeAm that functionally correspond to those in CeAl and receive predominant projections from the corresponding cell type in CeAl as shown in our model (Figure 8). This is consistent with a recent report with paired-cell recordings showing that inhibitory connections between neurochemically similar cell types (e.g., SOM(+) to SOM(+) cells) are stronger than those between different cell types in CeAl (Hunt et al., 2017). The mutual inhibitory connections between the two different types of SOM(+) and SOM(–) cells in CeA have been shown (Li et al., 2013) and would serve the opposing interactions of the negative circuit promoting negative emotion and the positive circuit that suppresses pain and negative emotion in central amygdala.

Chronic pain, a common neurological disease developed from persistent pain, is severely undertreated with no effective medications available at present and the comorbid emotional disorders such as anxiety and depression significantly contribute to the difficulties in mitigating this clinical problem (Wilson et al., 2001; McWilliams et al., 2003; Wiech and Tracey, 2009; Lumley et al., 2011; Bushnell et al., 2013; Duenas et al., 2016; Boersma et al., 2019; Zhou et al., 2020). Understanding of the interacting pain- and emotion-regulating brain circuits is of high significance and in urgent need. Pain signals from the peripheral are transmitted to the brain mainly along two pathways: the spino-parabrachial–limbic tract conveying the emotion-affective dimension of pain and the spino-thalamus tract carrying the sensory dimension of pain (Vogt, 2005; Shackman et al., 2011; Baliki and Apkarian, 2015; Yam et al., 2018). As a major part of the limbic system, CeA plays a critical role in pain-emotion integration and interaction through BLA of corticolimbic pathways (Bushnell et al., 2013; Han et al., 2015; Cai et al., 2018; Chiang et al., 2020). Our current results show two input-specific cell types in CeAl that are likely part of the opposing brain circuits for regulation of pain and emotion. It is thus proposed that, under persistent and chronic pain conditions, the pain-activated, increasingly sensitized activity in PBN-input-only cells of the negative circuit inhibits and outweighs adaptive activity of dual-input cells of the positive circuit descending from cortical structures, which are activated by pain stimuli as shown in previous human studies (Price, 2000; Zald, 2003; Vogt, 2005; Baliki and Apkarian, 2015), leading to negative emotion. Improved understanding of these regulating brain circuits and underlying cellular mechanisms would ultimately lead to development of novel drug targets and therapeutic strategies for the treatment of pain and associated emotional disorders.

## Data availability statement

The raw data supporting the conclusions of this article will be made available by the authors, without undue reservation.

## Ethics statement

The animal study was reviewed and approved by the Animal Care and Use Committee of The University of Texas MD Anderson Cancer Center.

## Author contributions

JG and ZP designed the research, analyzed the data, and wrote the manuscript. JG and YC performed the experiments. All authors contributed to the article and approved the submitted version.
